# Development of a Mobile Health Intervention with Personal Experiments for Smokers Who Are Ambivalent About Quitting: Formative Design and Testing

**DOI:** 10.2196/21784

**Published:** 2020-08-27

**Authors:** Jaimee L Heffner, Sheryl L Catz, Predrag Klasnja, Brooks Tiffany, Jennifer B McClure

**Affiliations:** 1 Fred Hutchinson Cancer Research Center Seattle, WA United States; 2 Betty Irene Moore School of Nursing University of California Sacramento, CA United States; 3 Kaiser Permanente Washington Health Research Institute Seattle, WA United States; 4 University of Michigan Ann Arbor, MI United States

**Keywords:** tobacco, nicotine, smoking, cessation, smartphone, motivation, mHealth, intervention, formative, development

## Abstract

**Background:**

The majority of cigarette smokers want to quit someday but are not ready to commit to long-term abstinence. However, available smoking cessation treatments are not well-suited to meet the needs of these ambivalent smokers. Low-cost, high-reach mobile health (mHealth) interventions may be a cost-efficient means of offering assistance to ambivalent smokers, yet there are currently no evidence-based options available for this group.

**Objective:**

The aim of this study was to develop and preliminarily evaluate the core content for an mHealth program targeting adult smokers who are ambivalent about quitting. The core content consisted of a series of “personal experiments” similar to those tested as part of a counseling intervention in prior work, including brief cognitive or behavioral tasks designed to boost readiness for changing smoking behavior.

**Methods:**

We conducted individual user interviews (N=3) to refine program content, and then conducted a one-arm pilot study (N=25) to assess user receptivity and the potential impact of the experiments on motivation and self-efficacy to quit or reduce smoking.

**Results:**

In user interviews, participants liked the concept of the personal experiments. Participants in the pilot study found a medium-fidelity prototype to be highly acceptable. After watching a brief orientation video that explained how the program works, most participants (80%, 20/25) indicated that it sounded interesting, primarily because it did not require any commitment to quit. All participants (100%, 25/25) completed all 7 experiments, including a 24-hour quit attempt, although not all were able to refrain from smoking for a full day based on qualitative feedback on the experiment. The mean rating of usefulness of the overall program was 4.12 (SD 1.09) out of 5, and the average rating of the difficulty of the experiments was 2.16 (SD 1.18) out of 5. At the last assessment point, 92% (23/25) of the participants indicated that they were more interested in either quitting or cutting back than when they began the program, and 72% (18/25) said that if the program had included a free trial of nicotine replacement therapy, they would have used it to try to quit smoking.

**Conclusions:**

This formative work confirmed that ambivalent smokers are willing to use and will remain engaged with an mHealth intervention that employs the novel concept of personal experiments to enhance their motivation for and ability to quit smoking. The addition of action-oriented treatment (self-help and free nicotine replacement therapy, quitline referral) could further support users’ efforts to stop smoking and remain quit.

## Introduction

### Background

According to the World Health Organization, tobacco kills approximately half of its users worldwide and is responsible for over 8 million deaths per year [1]. Helping people quit smoking is critical to reducing the human toll of tobacco use, but is also a difficult goal as most smokers are ambivalent about quitting. Approximately 70% of smokers in the United States report that they want to quit smoking someday, but they are not *currently* ready to quit nor actively seeking treatment [2]. This ambivalence makes it difficult to engage these individuals in nicotine dependence treatment programs. Consequently, reducing tobacco use on a population level will require new intervention strategies that can engage, motivate, and effectively assist ambivalent individuals in quitting smoking. Digital therapeutic interventions such as mobile health (mHealth) apps offer a potentially high-reach, effective strategy for achieving this important public health goal.

To date, no published trials have tested self-guided mHealth interventions designed for ambivalent smokers, despite strong rationale for doing so. The vast majority (81%) of US adults own a smartphone, including those with only a high school education (72%), those who make less than $30,000 a year (71%), and racial/ethnic minorities (Hispanic, 79%; Black, 80%) [3], which are also demographic groups with high smoking rates [4]. Further, rates of smartphone ownership are similar among smokers and nonsmokers [5]. Evidence from our preliminary work also suggests that smokers who are ambivalent about quitting—specifically, those who want to quit smoking someday, but not in the next 6 months—are quite receptive to mHealth interventions focused on smoking: 75% stated they would consider using a cessation app, 88% were interested in an app to help them reduce their smoking, and 91% were interested in an app that could help them decide “if , when, or how” to quit [6]. This suggests that, with appropriate intervention message-framing that takes their ambivalence into account, a self-guided mHealth intervention could reach and assist a sizable proportion of smokers who would not otherwise seek treatment. A particular advantage of mHealth in this context is that it does not require involvement of a treatment provider in contrast to other digital interventions that include ambivalent smokers as part of the target user group (eg, a provider-facilitated social media intervention targeting young adult smokers across all stages of change [7]). Thus, our aim was to create a novel, empirically validated, self-guided mHealth intervention that will be appealing to ambivalent smokers, able to keep them engaged over time, and ultimately assist them in quitting smoking.

### Intervening With Ambivalent Smokers

Research suggests that ambivalent smokers may benefit from standard, evidence-based treatment approaches (ie, behavioral and pharmacological interventions), but that they may also need to be introduced to these methods in a softer, more gradual manner than would be used with smokers who are already committed to quitting [8]. For example, we found that ambivalent smokers are willing to enroll in clinical trials when it is clear that the goal is to help them explore their willingness to quit or to answer questions about the quitting process, as opposed to asking for a commitment to stop smoking, and many go on to successfully quit [9,10]. Other studies have found that ambivalent smokers are receptive to a goal of smoking reduction, which in turn can increase quit rates, particularly when the intervention is paired with a stop-smoking medication [11-15]. This concept—that ambivalent smokers can benefit from the same evidence-based strategies used with smokers ready to quit, if framed appropriately—is consistent with West’s [16] PRIME theory, in which motivation is viewed as a dynamic, rapidly changing state rather than one that emerges slowly and in a staged manner. The implication is that interventions targeting ambivalent smokers should be responsive to rapid changes in motivation and able to support smokers’ changing needs and interests, while still focusing on similar goals and strategies that have been found to be effective for smoking cessation.

### Intervention Concept and Preliminary Work

We previously designed and pilot-tested a phone-based counseling program for smokers with depression, most of whom (69%) were ambivalent about quitting. A key component of the intervention was 9 weekly “experiments” that the participants were encouraged to try on their own between counseling sessions and to report back what they had learned. Each experiment was a short exercise designed to address cognitive restructuring and behavioral activation for mood management or to build self-efficacy for smoking cessation (eg, learning to delay smoking in response to urges, making a practice quit attempt) [17].

Findings from this study demonstrated the acceptability of using this approach to engage ambivalent smokers and support behavior change. We subsequently assessed ambivalent smokers’ reactions using a similar concept as a component of an mHealth intervention during user-centered design workshops. Participants in our user-centered design workshops were strongly in favor of trying what they retermed “personal experiments” to guide them through short, discrete activities that could help them learn the skills needed to change their smoking habits or to explore their interest in quitting. They particularly liked the idea of accessing these “personal experiments” through an mHealth app and suggested that users have the opportunity to earn points or rewards for completing each experiment. Gamification is a common request from smokers in our design work [6,18,19].

### Current Study

The goals of this study were to design and pretest a set of mHealth-delivered “personal experiments” for ambivalent smokers, using the experiments from our prior research as a guide, but modifying the topics and experiment structure (eg, duration) to work better as part of a self-guided program. Study findings are currently being used to refine the experiment concept for subsequent testing of the efficacy of this intervention as part of a more comprehensive mHealth app.

## Methods

### Prototype Development and Testing

#### Theoretical Foundation

The intervention concept was grounded in several prominent and complementary motivation and behavior change theories along with the empirically validated best-practice recommendations of the US Public Health Service (PHS) Guidelines for Treatment of Nicotine Dependence [20]. Consistent with the PRIME theory of motivation [16,21], the intervention acknowledges that motivation for behavior change is fluid and, in part, determined by one’s situational beliefs. Thus, the intervention assumes that motivation will fluctuate over time and targets smokers’ beliefs. In accordance with social cognitive theory [22-24], which forms the basis for cognitive behavioral therapy and many recommendations of the PHS Treatment Guidelines [20], we focus on promoting confidence (self-efficacy) and positive outcome expectations, since these are associated with success in quitting smoking. Toward this end, we used an approach to engage smokers in discrete experiments designed to shape their motivation and behavior change through traditional behavioral techniques such as successive approximation, reinforcement, and shaping. The experiments were also designed to teach the specific skills needed to cut back or quit smoking (eg, managing cravings) using techniques from both traditional cognitive behavioral therapy (eg, problem solving, stimulus control) and acceptance and commitment therapy (eg, values clarification, mindfulness, and other acceptance-based coping skills) [25-27]. We hypothesize that by engaging in the experiments, smokers will have successive mastery experiences that will build confidence and positive outcome expectations (ie, “I believe that I can control my smoking or I can quit when I am ready”), and in turn will encourage greater efforts for change, including making a quit attempt and ultimately quitting smoking. Drawing from Fogg’s [28] model for persuasive design, the intervention also recognizes that when people have low motivation for change (as is expected for ambivalent smokers), it is important that the behaviors they are asked to engage in are fairly simple (ie, require low ability) and that these behaviors need to be coupled with extrinsic triggers to prompt engagement (ie, reminder prompts).

**Figure 1:**
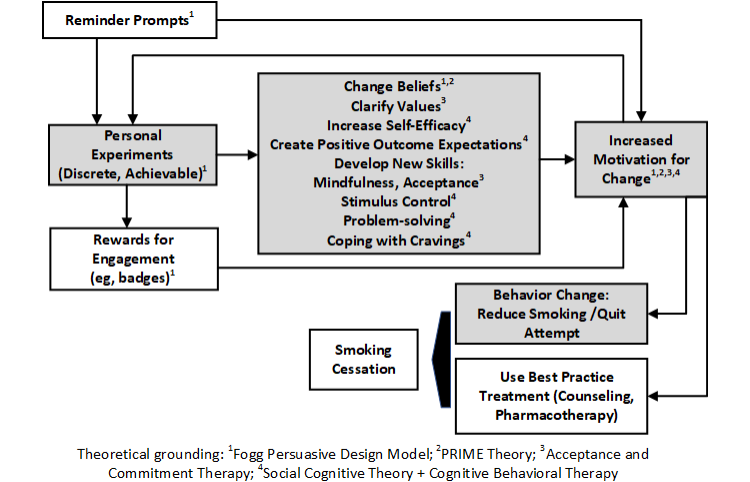
Conceptual model of the intervention.

To ultimately achieve smoking cessation, individuals may also need to utilize other treatment aids such as counseling or pharmacotherapy [20], but we believe that engaging in the personal experiments will increase the likelihood that this will occur. Figure 1 shows the theoretically based conceptual model of the intervention; items in grey reflect the core intervention elements being developed and evaluated in the present work.

#### Preliminary User Interviews

**Figure 2:**
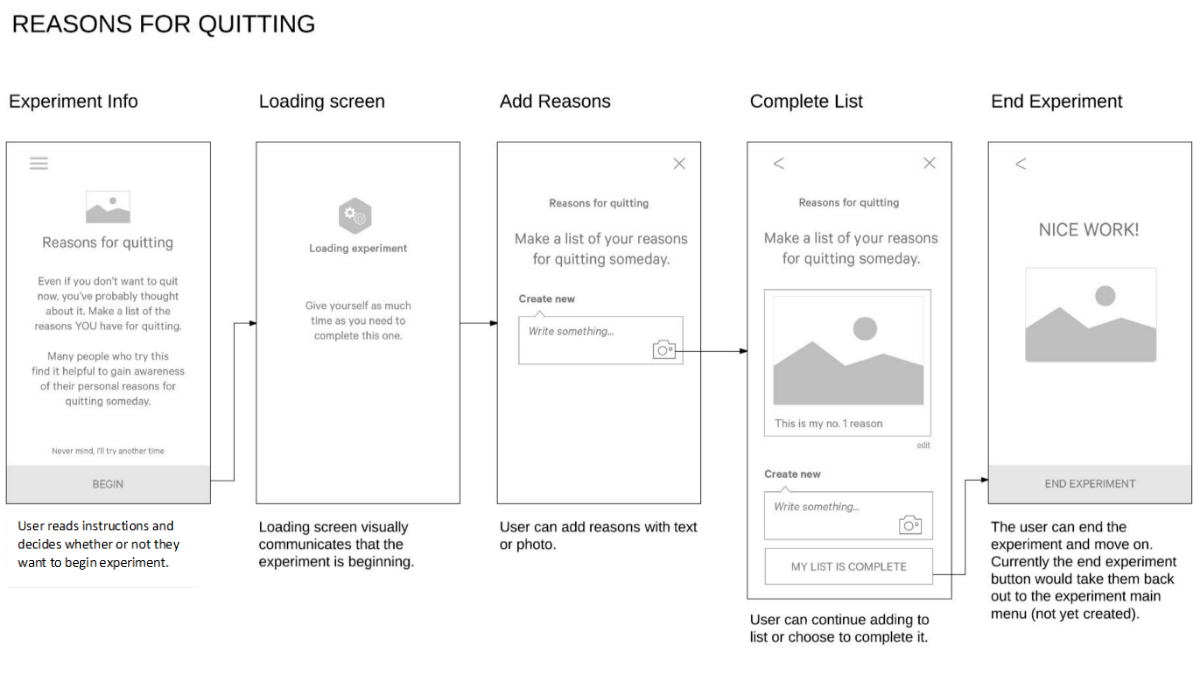
Example of a low-fidelity prototype of a personal experiment.

As a first step, we wanted to assess individuals’ reactions to the general experiment concept when presented as self-guided app-based exercises, and to collect feedback on several key issues that could help us refine the content and design of the experiments in the pilot study. For this purpose, we adapted 5 personal experiments from a prior study that used a similar experiment concept as part of a phone-based counseling program [17]. In that study, the counselor was able to explain each experiment to participants, who then had 1 week to complete each experiment. For an mHealth intervention, it is important that each experiment be self-explanatory, and we believe that a shorter time frame would be more optimal for keeping users engaged. Therefore, we created low-fidelity prototypes of 5 experiments that we believed were easy to understand and could be completed in less than 2 days (see Figure 2 for an example). We initially limited the number of experiments to 5 to reduce participant burden and maximize the time available during the interviews to more fully explore user reactions. The experiments included were specifically chosen to represent a range of goals and topics (ie, exploring motivation for quitting, tracking smoking behavior, changing smoking behavior or location), input options (ie, written comments, uploaded photos), duration (ie, experiments that could be completed in the moment and those that required taking action over a 24-48 hour period), and related design issues (eg, using progress indicators) that were considered pertinent to the final selection and design of the future experiments.

Five ambivalent smokers were screened as eligible to provide feedback on the initial prototype, but two cancelled and could not be rescheduled, leaving three final participants for this formative phase. Two of the three (67%) participants were women, all three were White, they were aged 40-46 years, reported smoking 5-10 cigarettes per day, and 2/3 (67%) reported household incomes under US $50,000/year (the other participant refused to answer). Each participant viewed a storyboard explaining the concept of the personal experiments as an mHealth intervention and were then walked through each of the experiment prototypes by a trained user-centered design researcher.

For each experiment, the participants were asked to identify any parts that were confusing, what they liked and disliked, whether they could see themselves trying the experiment, and what they anticipated would be the biggest challenges to their completing the experiment. They were also asked about the perceived helpfulness of a program that included these features; what, if anything, would help them stay engaged with the program over time; what other features they would like to see included; and whether they wanted to be able to share their experiment progress with others. The feedback from user interviews was then used to iteratively refine the basic intervention design and presentation prior to the pilot study.

#### Medium-Fidelity Prototypes

**Figure 3:**
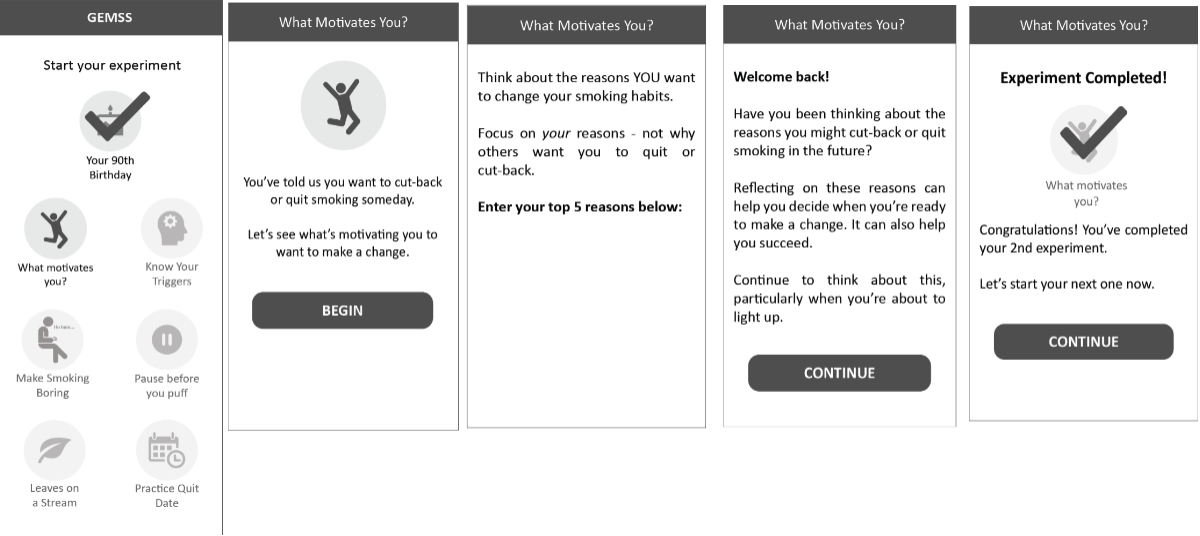
Medium-fidelity prototype of a personal experiment.

Using feedback collected from the user interviews, we designed a set of 7 personal experiments and then created a functional medium-fidelity prototype of the intervention program, including an initial program orientation and each personal experiment (see Table 1 for a summary of each and Figure 3 for sample content). Each experiment topic was chosen based on its theoretical or empirical utility for changing smoking-related attitudes, beliefs, or behaviors based on our prior research. The experiments were intentionally ordered based on the flow that we expect will maximize the intended therapeutic effects, starting with exercises designed to build or strengthen motivation for quitting, followed by experiments intended to teach coping or other behavioral skills needed to resist the urge to smoke in response to cravings and build self-confidence for making a quit attempt, and culminating in a 24-hour practice quit attempt.

An overview of each experiment and its intended cognitive or behavioral target is provided in Table 1. All experiments were designed to be completed either in the moment or within 48 hours.

**Table 1:** Personal experiments.

Experiment number	Title	Goal/skill targeted	Description
1	90^th^ Birthday	Clarify personal values.	User is asked to imagine giving a speech at their 90^th^ birthday party about what have been the most meaningful aspects of their life and to consider whether they want to be remembered as a smoker.
2	What Motivates You?	Identify reasons for quitting. Build motivation.	User is asked to make a list of all of the reasons that they want to quit smoking and review this list in response to cravings.
3	Know Your Triggers	Identify high-risk situations for smoking. Aid future problem-solving and coping.	User is asked to write down what they’re doing every time they have a craving to smoke.
4	Make Smoking Boring	Stimulus control. Make smoking less reinforcing.	User is asked to do no other activities (eg, no friends, coffee, TV) while smoking and to notice how it feels.
5	Pause Before You Puff	Learn to delay smoking in response to urges.	User is asked to wait 1 minute before each cigarette and to use that time to consider personal values or reasons for quitting, or to do nothing (ie, make smoking boring).
6	Leaves on a Stream	Learn mindfulness-based coping strategy. Learn to let urges pass without smoking.	User is asked to visualize thoughts as leaves on a moving stream and to practice nonjudgmentally observing these thoughts as they come and go (including thoughts about smoking) without acting on them.
7	Practice Quit Attempt	Make a practice quit attempt. No smoking for 24 hours.	User is asked to attempt to stop smoking for 24 hours and is encouraged to use nicotine replacement to assist with this challenge.

### Single-Arm Pilot Test

#### Participants

Twenty-five adult smokers were recruited to participate in a one-arm pilot test of the prototype intervention. Typically, randomized pilot trials of similar behavioral interventions include 25-30 participants per arm [29]; thus, 25 was viewed as an adequate sample size to assess the outcomes of interest in this formative study. Individuals were eligible if they were at least 18 years of age; smoked at least 100 cigarettes in their lifetime; reported any smoking in the last 7 days; were interested in quitting someday, but not currently trying to quit or planning to quit in the next month (ie, ambivalent about quitting); were comfortable reading and speaking in English; owned a smartphone; used apps on their smartphone at least once a week; and had a personal email account.

#### Procedures

Participants were recruited between September and November 2017 via advertisements on Craigslist in the following cities: Seattle, WA; Baltimore, MD; Columbus, OH; Atlanta, GA; and Oakland, CA. The cities were purposely chosen to obtain a geographically, racially, and socioeconomically diverse sample. To determine eligibility, potential participants were screened by telephone, following verbal consent to participate.

Eligible participants completed a baseline survey via telephone, either immediately following the telephone screening or in a later scheduled call. The survey assessed demographics, tobacco use, motivation to quit smoking, smartphone use, and interest in and experience with mHealth apps.

Participants were emailed a URL that allowed them to access the initial orientation and the first experiment from their smartphone. Study staff walked individuals through this content while on the phone to assess their reactions in real time and to ensure that they understood how to access and use the online prototype.

Starting on day 2, the remaining experiments were pushed to smokers one at a time via an emailed link. When the link was opened, smokers were taken to a mobile-optimized website, which mimicked the appearance and functionality of an mHealth app. Each experiment used a similar format, starting with a brief explanation of the experiment’s purpose, 1-2 screens explaining the action to be completed and encouragement to try it, and instructions that we would check back in 1-2 days to see how it went.

One to two days after each experiment was viewed online (which was monitored remotely), smokers were emailed a link that allowed them to complete a brief survey and then begin their next experiment. Surveys assessed motivation and self-efficacy for both quitting and reducing smoking, measured on a 5-point Likert-type scale where 1=“not at all” and 5=“very.” In addition to being more feasible to administer in a brief, repeated assessment protocol, the predictive validity of single-item measures of motivation and self-efficacy has been supported in studies focused on change in smoking and other substance use behaviors [30,31]. Participants also rated how helpful and difficult each experiment was using the same 5-point scale. With the exception of the first two experiments that involved a separate pre-experiment survey, the timing of the surveys allowed each to serve both as a postexperiment assessment and a pre-experiment baseline for the next experiment, enabling assessments of change over time in relation to each experiment.

The final survey contained additional questions about overall perceptions and impact of the program, including whether the program caused them to think differently about quitting or cutting back on smoking (yes/no) and, if yes, whether it made them more interested in quitting, more interested in cutting back, less interested in quitting, or less interested in cutting back. Participants were also asked if the program had provided a free 2-week supply of nicotine replacement therapy (NRT), whether they would have used it to try to quit. Response options were: “yes,” “unsure,” and “no, I would have saved them until I am ready to quit smoking.”

If participants failed to view an experiment within the planned 48-hour window, they were sent up to 4 email reminders. For the purpose of this pilot study, the entire series of experiments was designed to be completed in 2 weeks. Participants received US $75 for completing all experiments and a final follow-up survey.

The project was reviewed by the Kaiser Permanente Washington Human Subjects Review Board and deemed exempt from review due to its formative nature (ie, designed to develop a program rather than to produce generalizable knowledge).

#### Analyses

The majority of analyses for this formative work are descriptive, including means (SD) for continuous variables and frequencies and percentages for categorical variables. To assess the change in ratings of motivation and confidence, we used paired-sample *t* tests to compare pre- and postratings for each experiment, and we report the change score and 95% CIs for each comparison.

## Results

### User Interviews

Interviewees (N=3) responded positively to the intervention concept. They identified some aspects of the design that were confusing, including specific wording and iconography (eg, using an image of a camera to indicate the ability to upload photos). They also recommended several additional program features for future consideration, including allowing users to save their reflections about each exercise (eg, a journal), adding testimonials from other smokers, including statistics and information about smoking, and using gamification features to make the program more engaging and fun (eg, badges, challenges). Two of three participants were not interested in adding a social feature that would share their progress with others, citing a strong desire for privacy. Some of this feedback was incorporated into the medium-fidelity prototype (eg, dropping the ability to upload photographs). Other feedback that was out of the scope for the experiment concept (eg, adding a journal, testimonials, and reward badges) is being implemented and tested in an ongoing randomized pilot trial of the intervention.

### Single-Arm Pilot Test

#### Participant Characteristics

Among the 25 participants, 15/24 (63%) reported their race/ethnicity as nonwhite (11 Black, 1 Asian, 1 Mexican American, 2 with multiple responses, and 1 invalid response); 12% (3/25) of the participants were Hispanic, 64% (16/25) were men, 24% (6/25) had an education of high school or less, 68% (17/25) were employed, and 48% (12/25) had an annual household income less than US $45,000. Regarding tobacco product use, 56% (14/25) smoked cigarettes only and 44% (11/25) used another form of tobacco or nicotine product (eg, 7 used electronic cigarettes, 4 smoked cigars, and 3 used other tobacco products) in addition to cigarettes. The average number of cigarettes smoked per day was 17 (SD 11). More participants had Android phones (68%, 17/25) than iPhones (32%, 8/25). Only 2 of the 25 (8%) participants had ever used a smoking cessation app, although most (84%, 21/25) said that they would consider using one. All 25 participants said they would consider using an app that helped them decide if, when, or how to quit smoking. Nearly half (12/25, 48%) reported having experience using some other type of health app, with the most common being a physical activity app (9/25, 36%).

#### Receptivity 

After watching the brief program orientation that explained how the program works, most participants (80%, 20/25) indicated that it sounded interesting, primarily because it did not require a commitment to quit. All participants (100%, 25/25) completed all 7 experiments; 80% (20/25) completed these within 2 weeks, as planned, and 100% (25/25) within 1 month. Most of the participants (88%, 22/25) liked the order of the experiments presented. All participants (100%, 25/25) tried the 24-hour quit attempt, although not all were able to stay quit for a full day. The mean rating of usefulness of the overall program was 4.12 (SD 1.09) out of 5, and the average rating of the difficulty of the experiments was 2.16 (SD 1.18) out of 5. Regarding difficulty, feedback on the Practice Quit Date exercise highlighted the difficulty that some participants experienced in trying to go 24 hours without smoking, with some being unable to do so. For example, one participant, when asked what they disliked about the experiment, stated, “I did not like the fact that I was not able to quit for one day.” Another participant noted, “I tried using the tools that I learned from this study and to a certain degree it worked. I smoked less but I still smoked. I held out for most of one day, then I caved.” Participants’ comments also indicated that some were successful at the 24-hour abstinence goal. One participant stated, “I liked that I could go a whole day without smoking a cigarette. I thought I would have more withdrawal symptoms but I did not.” Another participant said, “Although it was very hard I did it!”

Helpfulness ratings (on a 1 to 5 scale where 1=not at all and 5=very helpful) for individual experiments are shown in Table 2 and ranged from 3.44 to 3.96, indicating a net positive rating for all experiments. The three experiments rated highest on helpfulness were Make Smoking Boring, Know Your Triggers, and Pause Before You Puff.

**Table 2:** Personal experiments and user receptivity outcomes.

Experiment	Title	Helpfulness, mean (SD)	Change in motivation to quit, mean (95% CI)	Change in confidence to quit, mean(95% CI)	Change in motivation to cut back, mean (95% CI)	Change in confidence to cut back, mean (95% CI)
1	90^th^ Birthday	3.44 (1.26)	–0.16 (–0.63, +0.31)	+0.08 (–0.35, +0.51)	–0.16 (–0.63, +0.31)	–0.20 (–0.63, +0.23)
2	What Motivates You?	3.60 (1.04)	–0.20 (–0.58, +0.18)	+0.20 (–0.18, +0.58)	–0.12 (–0.34, +0.10)	+0.20 (–0.31, +0.71)
3	Know Your Triggers	3.75 (1.23)	+0.52 (+0.20, +0.84)	+0.52 (+0.09, +0.95)	+0.56 (+0.07, +1.05)	+0.32 (–0.15, +0.79)
4	Make Smoking Boring	3.96 (1.17)	+0.52 (+0.23, +0.82)	+0.20 (–0.12, +0.52)	+0.32 (–0.03, +0.67)	+0.40 (+0.11, +0.69)
5	Pause Before You Puff	3.75 (1.29)	+0.04 (–0.21, +0.29)	+0.24 (–0.08, +0.56)	+0.00 (–0.32, +0.32)	+0.12 (–0.16, +0.40)
6	Leaves on a Stream	3.46 (1.18)	–0.32 (–0.71, +0.07)	-0.16 (–0.51, +0.19)	–0.28 (–0.61, +0.05)	+0.08 (–0.44, +0.28)
7	Practice Quit Attempt	3.68 (1.31)	+0.28 (–0.07, +0.63)	+0.32 (+0.06, +0.58)	+0.20 (–0.14, +0.54)	+0.29 (–0.11, +0.70)

#### Motivation to Quit

**Figure 4:**
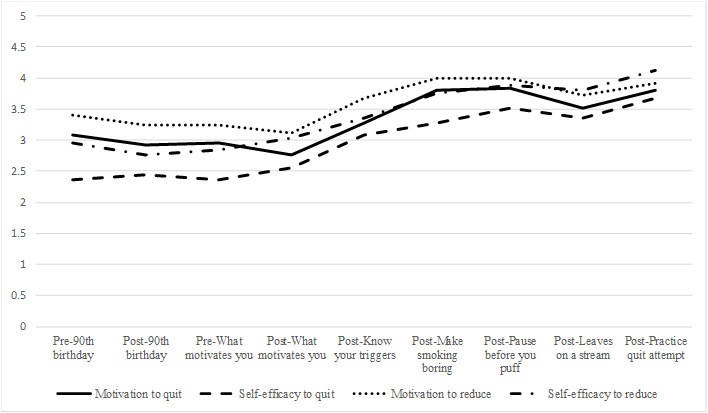
Change in motivation and self-efficacy across experiments.

At the last assessment point, 92% of the respondents (23/25) reported that trying the experiments made them think differently about quitting or cutting back, with a roughly even split between those who indicated that they were more interested in cutting back (11/25, 44%) and those who indicated that they were more interested in quitting completely (12/25, 48%). There was an average increase of 0.72 points on the 5-point motivation scale (95% CI +0.22 to +1.22) between the first pre-experiment assessment and the last postexperiment assessment. The impact of each experiment on motivation to quit is provided in Table 2, and Figure 4 shows the increases across experiments. The three experiments with the largest positive change in motivation to quit were Know Your Triggers, Make Smoking Boring, and Practice Quit Attempt.

#### Quitting Self-Efficacy

Confidence in ability to quit also showed an increase across the experiments (see Figure 4), increasing by over one point (+1.32, 95% CI +0.78 to +1.86) from the first to the last assessment. The three exercises with the largest increase in confidence to quit were Know Your Triggers, Practice Quit Attempt, and Pause Before You Puff (Table 2).

#### Motivation to Reduce Smoking

Motivation to reduce smoking increased across the experiments by one-half of a point (mean +0.52, 95% CI +0.12 to +0.92) from the first to the last assessment (Figure 4). The three exercises with the greatest increase in motivation to cut back were Know Your Triggers, Make Smoking Boring, and Practice Quit Attempt (Table 2).

#### Self-Efficacy to Reduce Smoking

Confidence in ability to reduce smoking increased across experiments (Figure 4) by an average of 1.17 points (95% CI +0.61 to +1.72) from the first to the last assessment. The three experiments associated with the greatest increases in confidence to cut back were Make Smoking Boring, Know Your Triggers, and Practice Quit Attempt (Table 2).

#### User Feedback on Adding Pharmacotherapy

Overall*,* 72% (18/25) of the participants said that if the program had included a free trial of NRT, they would have used it to try to quit; 20% (2/25) said that they were unsure if they would use NRT, and 8% (2/25) said they would save it until they were ready to stop smoking.

## Discussion

### Principal Findings

Results of this formative work provide proof-of-concept evidence that ambivalent smokers are willing to use and will remain engaged with a self-guided mHealth intervention using the concept of personal experiments to enhance their ability to quit smoking. These findings expand on our prior mixed methods study to assess the preferences and behavioral intentions of ambivalent smokers, in which we found a high rate of interest in using a digital health program where the messaging was framed specifically for ambivalent smokers (ie, smoking reduction or decision support to help them decide if, how, and when to give quitting a try) [6].

Motivation and self-efficacy, both for quitting and for reducing smoking, increased across the period of use, suggesting that the program impacted key cognitive targets. Because self-efficacy and motivation to quit are predictive of quit attempts and quit success [32,33], these findings are an encouraging signal of potential efficacy for supporting cessation among ambivalent smokers. Taken together with the strong indications of acceptability based on high perceived usefulness and high engagement with the program, these findings warrant continued development of the program as a novel method for engaging and assisting ambivalent smokers in a cessation program.

### Next Steps

Out of the 7 experiments, Leaves on a Stream was the only experiment that was identified as confusing based on the title and iconography (4/25), and after trying it, participants gave this experiment the lowest overall ratings. Based on this feedback, we plan to drop this experiment and replace it with an alternative mindfulness-based coping exercise. In addition, we plan to add more experiments designed to help users resist the urge to smoke in response to cravings (a critical skill for smoking cessation), include gamification features (ie, badge rewards), a journal feature to allow users to record lessons learned, and testimonials, in addition to pairing the program with best-practice treatment (self-help advice, NRT, and access to quitline counseling). These refinements are responsive to feedback from participants in the preliminary user interviews and in our other prior design work [6], as well as a body of research suggesting that providing active treatment to unmotivated smokers encourages quit attempts and improves cessation rates [34]. Inclusion of these components is also aligned with the PRIME theory of motivation [21]. Since motivation is dynamic, people may convert from ambivalence to readiness for action at any time. Thus, providing these resources will ensure that they have action-oriented support when it is needed.

### Limitations and Strengths

The small sample sizes in this research limit our ability to draw any conclusions about the generalizability of our findings. The lack of a control arm or long-term follow up in the pilot also prevents us from making strong assertions about the program’s impact on motivation, self-efficacy, and behavior change. Self-report data are also subject to social desirability bias. Although these limitations should be considered when interpreting the results, the methods are appropriate for this formative stage of design.

Strengths of this work include a demographically diverse participant sample, a rigorous assessment strategy that included pre-post evaluation on key constructs of interest immediately prior to and following each experiment, and a high pilot retention rate. Given the dearth of knowledge about how best to utilize digital health technologies to support behavior change among people ambivalent about quitting, our target population is also a study strength.

### Conclusions

This work highlights both the need and the promise for interventions targeted to smokers who are ambivalent about quitting. If found to be effective in future work, the planned intervention could provide an attractive new option for ambivalent smokers as well as for employers, quitlines, and health care organizations, none of whom currently has evidence-based options available to offer this group. The results may also be relevant when designing mHealth interventions for people not yet ready to commit to other types of health behavior change (eg, physical activity, dietary intake, alcohol use), as the concept of personal experiments may be a useful strategy for engaging users and promoting action without requiring a commitment to change.

## Abbreviations

mHealth: mobile health
NRT: nicotine replacement therapy
PHS: Public Health Service
